# Point Mutations in *Helicobacter pylori*'s *fur* Regulatory Gene that Alter Resistance to Metronidazole, a Prodrug Activated by Chemical Reduction

**DOI:** 10.1371/journal.pone.0018236

**Published:** 2011-03-25

**Authors:** Sung Sook Choi, Peter T. Chivers, Douglas E. Berg

**Affiliations:** 1 Department of Molecular Microbiology, Washington University Medical School, St Louis, Missouri, United States of America; 2 Department of Biochemistry and Molecular Biophysics, Washington University Medical School, St Louis, Missouri, United States of America; University of Hyderabad, India

## Abstract

**Background:**

*Helicobacter pylori*'s Fur regulatory protein controls transcription of dozens of genes in response to iron availability, acidity and oxidative stress, and affects the vigor of infection and severity of disease. It is unusual among Fur family proteins in being active both when iron-loaded and iron-free.

**Metholodolgy/Principal Findings:**

We tested if *H. pylori fur* mutations could affect resistance to metronidazole (Mtz), an anti-*H. pylori* prodrug rendered bactericidal by chemical reduction. Point mutations were made by PCR in DNA containing *fur* and a downstream chloramphenicol resistance gene, and were placed in the *H. pylori* chromosome by transformation of a *fur-*deletion (*Δfur*) strain. Several substitutions affecting *H. pylori* Fur's ∼10 residue N terminal arm, which has no counterpart in prototype (*E. coli*-type) Fur proteins, increased Mtz resistance, as did mutations affecting the region between DNA binding and dimerization domains. Three types of mutations decreased resistance more than did Δ*fur*: substitutions affecting the N-terminal arm; substitutions affecting the metal binding pocket; and nonsense mutations that resulted in a truncated Fur protein with no C-terminal dimerization domain. Most metal binding pocket mutations were obtained only in *fur* genes with additional inactivating mutations, and thus seemed deleterious or lethal because they.

**Conclusions/Significance:**

These results establish that *H. pylori* Fur's distinctive N terminal arm is functional, and more generally illustrate that point mutations can confer informative phenotypes, distinct from those conferred by null mutations. We propose that *fur* mutations can affect Mtz susceptibility by altering the balance among Fur's several competing activities, and thereby the expression of genes that control cellular redox potential or elimination of bactericidal Mtz activation products. Further analyses of selected mutants should provide insights into Fur interactions with other cellular components, metabolic circuitry, and how *H. pylori* thrives in its special gastric niche.

## Introduction

The gastric pathogen *Helicobacter pylori* chronically infects the stomachs of billions of people worldwide. It is highly specific for gastric epithelial cell surfaces and a narrow band of overlying mucous, an inherently unstable niche that is hostile to other microbial species [1]–[3]. *H. pylori* infections typically start in early childhood, can last for life if not treated, and constitute a major cause of gastric and duodenal ulcer diseases and gastric cancer. Persistent infection is thought to depend on a constellation of quantitative factors – prominent among them, *H. pylori*'s abilities: (i) to provoke low level tissue damage and inflammation and the release of nourishing metabolites, without destroying the gastric epithelium on which *H. pylori* depends; (ii) to cope with inflammation-associated oxidative stresses and transient exposure to stomach acid; (iii) to acquire iron and other micronutrients needed as metalloprotein cofactors and protein structural components, while avoiding the toxicity of these metals when they are in excess or not properly sequestered; and (iv) to swim away from acidic environments and toward the near-neutral, nourishing epithelial surface [1]–[7].

Each of these activities is affected or controlled in part by the *H. pylori* Fur protein, which belongs to a widespread family of transcription regulators whose members have been most studied in terms of controlling iron uptake and utilization [4]–[6], [8]. Prototype Fur proteins, such as that of *Escherichia coli*, act as simple repressors when complexed with ferrous iron and are inactive when iron-free. Their iron-bound forms (Fe-Fur) directly block transcription of some target genes (e.g., for iron uptake) and increase transcription of other genes (e.g., for iron storage), whereas their iron-free forms (Apo-Fur) are inactive. In these cases, positive regulation by Fe-Fur is indirect – via repression of transcription of a gene whose product, in turn, represses target genes.

**Figure 1:**
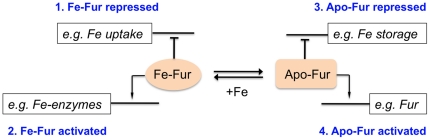
Positive and negative transcriptional regulation by *H. pylori* Fur protein when complexed with iron (Fe-Fur) and when free of iron (Apo-Fur). Based on refs 4–6.

**Figure 2:**
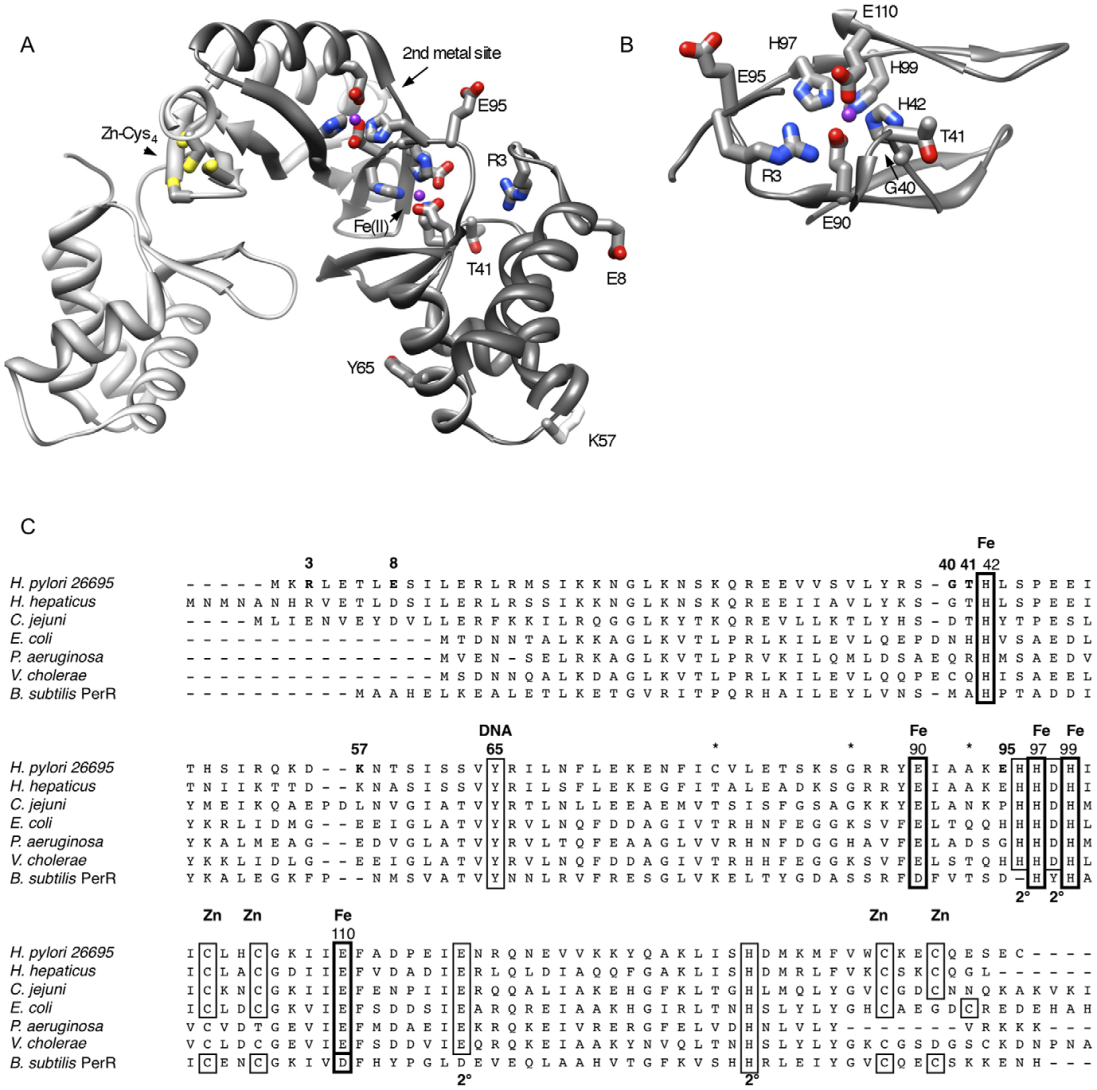
Mutational changes in *H. pylori* Fur that affect Mtz resistance in distinct regions of the protein. A) Structure of the Zn-loaded *H. pylori* Fur dimer [9] (PDBID 2XIG). This shows mutated residues and those of the Fe-binding pocket (H42, E90, H97, H99, E110), secondary (2°; H96, D98, E117, H134) and structural Zn-sites (C102, C105, C142, C145) [9]. This image was generated using Chimera [10]. B) A rotated view (∼90°) of the Fe-binding site of a Fur monomer subunit. Protein side chains are colored by atom (red, oxygen; blue, nitrogen; yellow, sulfur; and gray, carbon). Metal ions where visible are colored purple. The positions of three classes of mutations identified in this study are indicated: DNA-binding domain – E8, Y65, K57; DNA binding domain-iron binding pocket/dimerization domain interface – R3, G40, T41, E95; and Fe-binding pocket (see numbering above). C) Sequence alignment of *H. pylori* strain 26695 Fur protein with Fur sequences from other selected bacterial species. Residues that were mutated in this study are indicated with bold numbers and letters. Conserved metal- or DNA-binding ligands are boxed and their functional roles are indicated above the alignment. The primary Fe-site residues (bold outline) are based on the structure of Zn-loaded *HpFur*, but have not been demonstrated crystallographically for any Fur protein. The structural Zn-site is not conserved amongst along Fur family members.

The Fur protein of *H. pylori* seems more complex functionally than the prototypes: Apo-Fur and Fe-Fur each bind cognate regulatory site DNAs; and each represses transcription of certain genes and apparently stimulates transcription of others. Furthermore, *H. pylori* Fur autoregulates its own synthesis – inducing synthesis when iron is limiting by binding to one regulatory DNA sequence, and repressing synthesis when iron is abundant by binding to a nearby sequence (Fig. 1) [4]–[6]. We hypothesize that *H. pylori* Fur has two distinct active conformations: Fe-Fur (Fig. 2A), which binds to one set of operator DNA sequences; and a distinct Apo-Fur form, which binds to other operator sequences (see for example ref 11). Positive regulation by *H. pylori* Fur is thought to be direct [4]–[6] – formally equivalent to that by λ CI repressor [12], although the possibility of indirect control is raised again by the finding of many small non-coding regulatory RNAs in *H. pylori* cells [13]. X ray crystal structures of several Fur and Fur-type regulatory proteins complexed with zinc or manganese have been determined [9], [11], [14]–[17].

Several mutational changes in *H. pylori* Fur proteins that affect parameters such as repression mediated by Fe-Fur or Apo-Fur, iron loading, sodB (superoxide dismutase) expression and metronidazole (Mtz) resistance and/or protein dimerization were described recently [18], [19]. Although the structure of a Zn-bound form of a mutated but still active form of *H. pylori* Fur has been determined recently [16], much still remains to be learned about this protein's functionally important residues and conformations, especially those of Apo-Fur and the form(s) that mediate responses to acid or oxidative stress.

The present experiments were begun after finding a mutation in *fur* gene codon 3 (change of Arg to Ile; “*furR3I*”) in an *H. pylori* strain that had undergone multiple steps of mutation and selection for increased resistance to metronidazole (Mtz) [20]. This mutation affected *H. pylori* Fur's N terminal arm, a ∼10 amino acid long segment with no counterpart in Fur proteins that had been structurally characterized previously, and whose role, if any, had not been assessed previously. The potential importance of N-terminal arms, however, had been well documented in early studies of phage λCI and *E. coli* AraC repressor proteins [21]–[23]; and N-terminal arms similar in length to *H. pylori* Fur's, but divergent in sequence, are found in Fur proteins of *H. pylori*-related species (Fig. 2C). An analogous N-terminal arm is also found in *H. pylori*'s nickel-responsive NikR regulatory protein, a feature whose presence and composition also varies widely among related proteins [24], and that affects DNA binding specificity [25]. Accordingly, we hypothesized that *H. pylori* Fur's N-terminal arm is functional, and that the appearance of the *furR3I* mutation stemmed from its enhancement of Mtz resistance. Mtz exposure is mutagenic [26] however, and a *fur* mutation had appeared in only one of the two highly resistant lineages that were analyzed [20]. Hence, an alternative interpretation was that *furR3I* might be a bystander, and that the observed increase in Mtz resistance had been caused by mutation at another unknown locus.

It is with this background, interest in novel features of multifunctional regulatory proteins such as Fur and survival strategies of niche-specialists such as *H. pylori*, and with the long-range goal of better understanding *H. pylori* Fur's structure, function and regulatory circuitry, that we carried out the mutational analysis of *H. pylori fur* described below.

## Results

### The *furR3I* point mutation and a *fur* deletion each affect Mtz resistance, but differently

**Figure 3:**
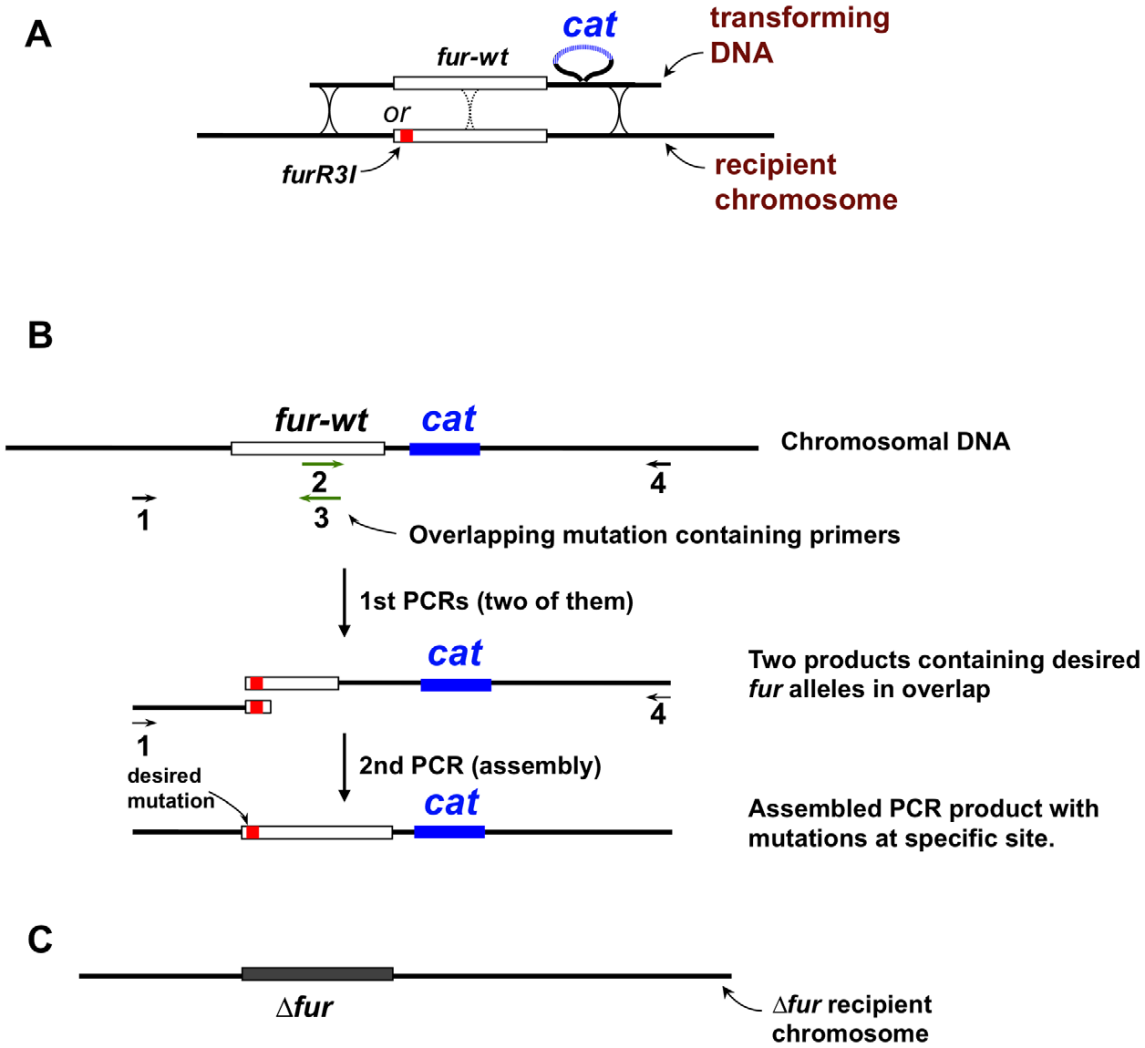
Strategy for efficient mutational analysis of *H. pylori* Fur. A) Transformation based strategy for placing any gene or mutation of interest at a predetermined place in the *H. pylori* chromosome. Recipient strains used here contain an *aphA* (amino phosphotransferase; kanamycin resistance) gene in place of *fur*. Homologies of at least ∼0.5 kb between transforming DNA and the recipient chromosome both upstream of *fur* and downstream of the inserted *cat* (chloramphenicol acetyl transferase, resistance) gene allow efficient recombination and recovery of *fur* alleles by selection for chloramphenicol resistance. B) Two step strategy for directed sequence change within *fur*. Primers 2 and 3 overlap and cover the site to be changed; primers 1 and 4 lie upstream and downstream, respectively, of the *fur-cat* gene pair. Initial PCRs with primers 1 and 2, and separately with primers 3 and 4, generate two PCR products that overlap to an extent determined by the sequences of primers 2 and 3 (typically ∼40 bp in our studies) and that contain the intended mutant allele(s). A second PCR with the two products of the first PCRs and primers 1 and 4 generates a product suitable for transformation into a *Δfur* recipient, as in panel A. An equivalent strategy was used to generate more complex multi-mutant *fur* alleles by splicing complementary singly mutant segments of *fur* together; and also to generate a deletion with defined endpoints such as *furΔ2*–*7*, which was made with long primers whose 5′ and 3′ portions corresponded to sequences flanking the segment to be deleted. The sequences of primers used for PCR amplification, mutation and sequencing are listed in Table S1.

To critically test if the *furR3I* allele does increase Mtz resistance, a chloramphenicol resistance gene (*cat*) was inserted downstream of a wild type (*wt*) *fur* gene by a direct PCR method [27], [28]. The resultant PCR product was used to transform *H. pylori* strain M1.5 (Fig. 3A), which contains *furR3I* and also four other mutations that had appeared earlier in our multistep selection for high level Mtz resistance (in genes *rdxA*, *frxA*, *ribF* and *mdaB*) [20]. Dilution and plating tests showed that single cells of strain M1.5 reproducibly formed colonies with 100% efficiency on agar medium containing 230 µg of Mtz/ml, but were killed (∼1% survival) on agar containing 250 µg of Mtz/ml (phenotype designated 230R,250S) [20], [29]. Chloramphenicol resistant (Cam^r^) transformants of strain M1.5 were selected, and *fur* genes of several of them were PCR amplified and sequenced. Those transformants that had received the *fur-wt* allele had a 190R,220S phenotype, and were less Mtz resistant than isogenic Cam^r^ siblings that had retained *furR3I* (230R,250S). These quantitative differences in Mtz resistance phenotypes were seen reproducibly in side-by-side comparisons on the same petri plates, and in repeated efficiency of plating tests. This outcome established that *furR3I* does indeed contribute to strain M1.5′s high level Mtz resistance.

**Figure 4:**
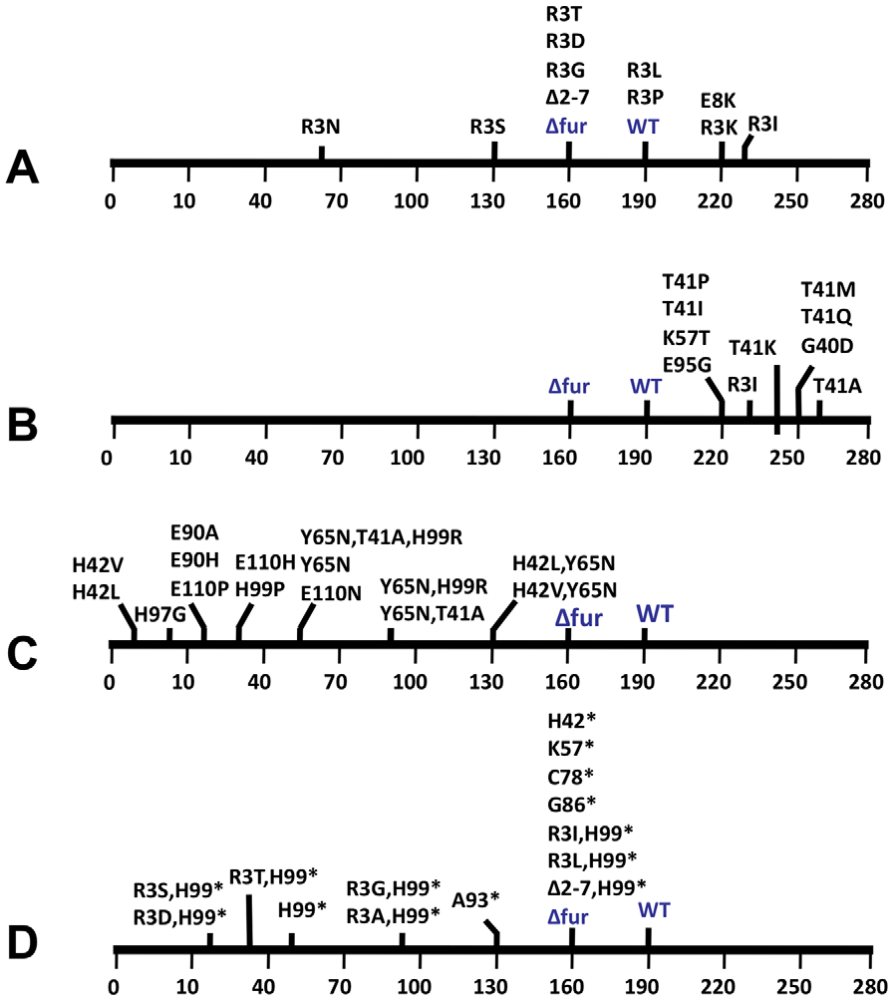
Graphic presentation of effects of representative *fur* alleles on Mtz susceptibility. The *fur* substitution mutations indicated here are named using the one letter code according to amino acid in wild type, its numerical position in the *H. pylori* Fur protein, and the amino acid encoded by the mutant allele. For example, *furR3I* indicates a mutation at codon 3 that changes arginine to isoleucine at Fur position 3. The horizontal scales represent the highest level of Mtz in µg per ml in agar medium (0 to 280) that allows 100% efficiency of colony formation by cells of a given derivative of strain M1.5 (mutant in *rdxA, frxA, ribF, mdaB*), defined by its *fur* allele. Thus, the *furR3I* strain forms colonies with 100% efficiency on medium with 230 µg Mtz/ml, and with less than 1% efficiency on medium with 250 µg Mtz/ml (phenotype designated 230R,250S in text). Mtz concentrations in media used for these analyses were 0, 3, 5, 8, 16, 32, 64, 50, 90, 130, 160, 190, 220, 230, 250, 260 and 280 µg/ml. A) Effects on Mtz resistance of sequence changes in *H. pylori* Fur's N terminal arm shows that this arm is important functionally. B) Point mutations that increased resistance obtained by random PCR-based mutagenesis of the entire *fur* gene, transformation as in Fig. 3, and initial selection for colony formation on medium with chloramphenicol and 200 µg of Mtz per ml. C) Effects of changes in residues likely to be part of the iron binding pocket. The majority of transformants containing changes at these positions also contained inactivating (generally frameshift) mutations elsewhere in the *fur* gene, suggesting that most such putative iron binding pocket changes were deleterious or lethal. The subset that were recovered without additional changes (depicted here) are thus likely to be leaky alleles, possibly still able to bind iron with low efficiency. D) Decrease in Mtz resistance caused by *H. pylori* Fur truncation at positions indicated by asterisks (nonsense (stop) codon mutations). Especially noteworthy are the extreme Mtz sensitizations caused by several double mutant alleles, in particular furR3T,H99* and furR3D,H99*, which are far more severe than the sensitizations caused their component single mutant alleles. Not depicted in the Figure were also several dramatic Mtz sensitization phenotypes conferred by *fur* alleles with unintended frameshift mutations near codon 110 and that had emerged from an attempt to change E110. Phenotypes of 64R, 32R, and 32R were obtained in M1.5 derivatives whose altered C terminal amino acid sequences from position 109 were IRFCRP*, IQFARP* and HSLCRP*, respectively (* designates stop codon). The sequence of the corresponding region of Fur wild type is IEFADPE.

In parallel, PCR products were generated in which the *fur* gene was replaced by a kanamycin resistance determinant (*aphA*) (Δ*fur-aphA* allele), or simply deleted in PCR products containing a downstream *cat* gene (Δ*fur-cat* allele). Kan^r^ or Cam^r^ transformants of strain M1.5 made with these constructs were less Mtz resistant (phenotype 160R,190S) than were those containing the *fur-wt* allele (190R,220S) (Fig. 4A). The finding that *Δfur* decreases resistance whereas *furR3I* increases resistance, relative to *fur-wt*, further emphasizes that *H. pylori* Fur protein's N-terminal arm is functional.

### Mutations in N-terminal arm can variously decrease or increase resistance

We had found that reference strain 26695, the wild type ancestor of strain M1.5, is exquisitely sensitive to low levels of Mtz (1R,3S in phenotype) [29], [30], and that strain M1.5′s high level Mtz resistance could be increased further by additional cycles of selection on agar with higher levels of Mtz (D Dailidiene and DE Berg, unpublished). This outcome and the different resistance levels conferred by *Δfur*, *fur-wt* and *furR3I* alleles in strain M1.5 indicated that this strain would be well suited for scoring phenotypic effects of other *fur* alleles over a large dynamic range. We therefore used the *Δfur-aphA* derivative of M1.5 as a recipient strain for *fur* DNA transformation and analyses described below.

Additional mutations at codon 3 were made using the two-step PCR protocol diagrammed in Fig. 3B to learn if all changes at this position that affected Mtz resistance would increase it. A first PCR was carried out with primers designed to generate a near-random set of codon 3 mutations (primers #2 and #3) in combination with upstream and downstream flanking primers (#1 and #4, respectively). This generated two sets of PCR products that overlapped by 44 bp, and that each contained many codon 3 changes. A second PCR using these two products and only the flanking primers (#1 and #4) generated a population of full length *fur*,*cat* DNA products that was then used to transform strain M1.5 *Δfur-aphA* (Fig. 3C). All Cam^r^ transformants were Kan^s^, as expected of replacement of the *Δfur-aphA* allele. Eight transformants carrying new codon 3 alleles but no other mutations elsewhere in *fur* were identified by DNA sequencing, and were tested quantitatively for levels of Mtz resistance. Two mutations, *furR3S* and *furR3N*, decreased Mtz resistance more than did the *Δfur* alleles – at the limit, conferring a 64R,90S phenotype, in contrast to the 160R,190S phenotype conferred by *Δfur*); one mutation, *furR3K*, increased resistance (220R,230S) almost as much as did the original *furR3I* mutation (230S,250R), and five others conferred levels of resistance similar to those of the *Δfur* or *fur-wt* alleles (*furR3G, R3D, R3T* and R3P, R3L, respectively) (Fig. 4A). A mutation of *fur* codon 8 (*furE8K*) had been found in a separate direct selection for increased Mtz resistance (D Dailidiene and DE Berg, unpublished). DNA containing the *furE8K* allele was tagged with the downstream *cat* gene (Fig. 3A) and used to transform strain M1.5 *Δfur-aphA*. Like *furR3I*, this *furE8K* allele conferred a 220R,230S phenotype in the strain M1.5 background, indicating that Fur N-terminal arm position 8 is also important functionally. A deletion of *fur* codons 2 through 7 (Δ2–7 in Fig. 4A) was made similarly. It conferred a Mtz resistance phenotype equivalent to that of the total *fur* gene deletion (*Δfur*) (Fig. 4A). Collectively, these results show further that *H. pylori* Fur's distinctive N terminal arm is functional. Because some position 3 changes increased resistance, whereas others decreased resistance more than did simple null mutations, we propose that the Fur's N terminal arm can adopt two different structures and actions, only one of which is indicated in the recently determined structure of zinc-loaded Fur protein [9] presented in Fig. 2.

### Induction and direct selection of *fur* mutations that affect Mtz resistance

Mutations anywhere in *fur* that increased Mtz resistance were also sought. A DNA fragment containing *fur-wt*, a downstream *cat* gene and flanking sequences (Fig. 3A) was PCR amplified in MnCl_2_-containing buffer to stimulate DNA synthesis errors, and the product was used to transform strain M1.5 *Δfur-aphA*, as described above. Four transformants selected only for chlorampenicol resistance contained frameshift mutations in *fur*, had resistance phenotypes equivalent to those of isogenic *Δfur* strains (160R 190S), and were not studied further. Next Cam^r^ transformants were selected for resistance to 200 µg of Mtz/ml, a concentration sufficient to kill >99% of isogenic *fur-wt* Cam^r^ cells. Of the *fur* genes from 20 transformants sequenced, ten contained single substitutions, six of which affected just one position in Fur protein (position 41); the other four transformants contained single mutations affecting protein positions 40, 41, 57 and 95.

Since Mtz treatment is mutagenic [26] and these transformants had been selected on Mtz-containing agar, we tested if their *fur* alleles did indeed cause increased Mtz resistance by transforming them into strain M1.5 *Δfur-aphA*, with selection only for the linked chloramphenicol resistance marker. Tests of transformants verified that each mutation had indeed increased Mtz resistance, relative to the *fur-wt* allele (Fig. 4B). Based on the Fur protein structure (Fig. 2) [9], changes at positions 40, 41 and 95 should affect the interface between the N terminal DNA binding and C terminal dimerization domains and/or the iron binding pocket's conformation or flexibility; the change at position 57 should affect a DNA binding interface.

### Iron binding pocket residues that affect Mtz resistance or viability

Given that both the Fe- and Apo- forms of *H. pylori* Fur have regulatory activity [4], [5] (Fig. 1), five residues that seemed likely to affect the conformation of the metal-binding pocket and/or make direct contact with bound iron (Fig. 2B) were targeted next for mutagenesis. We began by attempting to make a strain M1.5 derivative with *furH99R*, an allele that should be equivalent to the Salmonella *furH90R* allele that had resulted in iron-independent Fur-mediated regulation of acid resistance [31 29]. However, 10 of 12 Cam^r^ transformants of strain M1.5 *Δfur-aphA* generated using PCR products made with H99R-specific primers (as in Fig. 3) contained frameshifts; one contained a scrambled sequence; and one contained the original wild type sequence. None contained only the desired H99R change. Similarly, each of three Cam^r^ transformants made with the same PCR products, but using as recipient a *Δfur-aphA* derivative of wild type strain 26695 (the Mtz^s^ ancestor of strain M1.5; wild type alleles of genes *rdxA, frxA, ribF, mdaB*; ref. 20) also contained a frameshift or scrambled sequence. Collectively, these data suggested that this *furH99R* allele is deleterious or lethal in *H. pylori*, independent of the status of four genes likely to affect cellular reductive potential.

In parallel experiments, we generated a *furY65N* allele, where residue Y65 in *H. pylori* Fur corresponds to the critical Y55 component of a DNA binding helix of *E. coli* Fur [32]. Both *furT41A* and *fur-wt* containing DNAs were used as PCR templates (Fig. 3B). The *furY65N* allele caused severe Mtz sensitization in strain M1.5, in both *furT41*-wild type and *furT41A* (resistance-enhancing) contexts: 64R,90S and 90R,130S phenotypes, respectively. This contrasts with the 190R,220S and 260R,280S phenotypes of isogenic strains containing a non-mutant *furY65* site and the 160R,190S phenotype of the isogenic *Δfur* strain (Fig. 4C). Having found *furY65N* to decrease Mtz resistance, we next generated *furH99R,Y65N* double and *furH99R,T41A,Y65N* triple mutant alleles. The triple mutant was made first, using a *furY65N,T41A* template and *furH99R-*containing primers (Fig. 3); 10 of 11 Cam^r^ transformants contained the desired three mutations with no additional mutations in *fur*. The double mutant was made similarly, starting with triple mutant DNA as PCR template, and primers with wild type sequence covering codon 41; each of the two Cam^r^ transformants analyzed contained the desired *furY65N,H99R (fur* codon *41-wt)* allele, with no additional mutations in *fur*. These results indicate that *furH99R* 's deleterious or lethal impact can be compensated by the *furY65N* mutation, probably because it diminishes Fur protein-DNA binding. Strain M1.5 derivatives carrying the *furH99R,Y65N* double mutant allele exhibited a 90R,130S (highly Mtz-sensitive) phenotype. Paradoxically those that also carried the *furT41A* mutation, which enhanced resistance in other contexts, were even more Mtz-sensitive (50R,64S).

A more general search for non-lethal iron binding pocket changes was carried out using pairs of mutagenic primers designed to generate random amino acid replacements of critical histidine and glutamic acid residues (H42, H97, H99, E90 and E110; Fig. 2), using *fur-wt cat* DNA as template and strain M1.5 *Δfur-aphA* as the transformation recipient (Fig. 3). The *fur* genes of 62 transformants selected solely for chloramphenicol resistance were sequenced (average ∼12/targeted site). Collectively, only nine amino acid replacement mutations were obtained at these various sites with no other unintended changes (Fig. 4C, below). Rather, most transformants contained additional changes, variously frameshifts (27/62), scrambled sequences (9/62) and stop codons (2/62), or were not mutant (11/62). This low yield of simple single mutations suggested that most changes affecting *H. pylori* Fur's iron-binding pocket were deleterious or lethal, and thus recoverable in transformants only if the *fur* gene had been inactivated.

Each of the simple iron binding pocket mutations that we did obtain caused severe Mtz sensitization (ranging from 3R,8S to 64R,90S, depending on allele) (Fig. 4C). The two most extreme alleles, *furH42V* and *furH42L*, also caused slower growth on Mtz-free agar (colony formation from single cells in three rather than two days), whereas the other seven (*furE90A* or *H*, *furH97G*, *furH99P*, or *furE110H, P* or *N*) had no obvious effects on colony size or growth rate. Further experiments will be needed to test if the extreme Mtz susceptibility and the poor growth conferred by these *furH42* mutations stem from loss of Fur's iron-dependent activities, perhaps without much impairment of Apo-Fur activities.

Although mutations *furE90A*, *E110N* and *Y65N* each caused marked Mtz sensitization (16R-50R range), double mutants containing *furY65N* and also *furE90A* or *Q* or *E110N, R* or *P* each conferred a *Δfur*-like 160R,190S phenotype (data not shown). Possible explanations include degradation of the double mutant Fur proteins or altered balance of Apo-Fur and Fe-Fur activities.

### Mtz sensitization caused by *H. pylori* Fur truncation

A nonsense mutation in *fur* codon *H99* (designated furH99*), obtained by PCR mutagenesis (Fig. 3), caused severe Mtz sensitization (50R,64S, in contrast to 160R,190S conferred by *Δfur*) (Fig. 4D). In addition, three alleles obtained by mutating *fur* codon *E110* each contained nearby frameshift mutations that resulted in Fur protein truncation at protein position 115 and caused Mtz sensitization (Fig. 4). In contrast, nonsense mutations at codons 42, 57, 78 and 86 each conferred a 160R,190S phenotype, equivalent to that of *Δfur* alleles. A codon 93 nonsense mutation conferred an intermediate 130R,160S phenotype (Fig. 4D). The similar phenotypes conferred by a *Δfur* allele and by nonsense mutations that caused protein truncation at or before position 86 suggest that Fur remnants of ≤86 amino acids lack all regulatory activity or are degraded. The greater sensitization caused by truncation at codons 93, 99 and 115 would then be explained if the longer Fur remnants retain some Apo-Fur DNA binding activity (despite an inability to dimerize or bind iron effectively) and an ensuing imbalance in Fur-regulon gene expression.

The double mutant alleles furR3S,H99* and furR3D,H99* each conferred a 16R,32S phenotype, which is lower than the resistance phenotypes conferred by any of these three *fur* mutations alone (Fig. 4C,D). This outcome indicates that the *H. pylori* Fur N terminal arm's critical role does not require interaction with the C terminal dimerization domain, and suggests that the arm and dimerization domains have separate (additive) effects on *H. pylori*'s regulatory circuitry. In addition, the alleles furR3I,H99* and furR3L,H99* conferred a *Δfur*-like (160R,190S) phenotype. Perhaps these latter two changes of residue 3 cause Fur protein inactivation or degradation when in a Fur remnant context; this would be distinct from the changes in gene regulation that they cause when full length Fur proteins.

### Epistasis and background genotype impact on Fur-determined phenotypes

Loss of function mutations in the related *rdxA* and *frxA* nitroreductase genes are responsible for the first two steps in development of Mtz resistance in most wild type Mtz^s^ *H. pylori* strains [29], [30], and restoration of a functional *rdxA* gene in strain M1.4 (*fur* ^+^ parent of M1.5 used here) restored the original Mtz sensitive phenotype nearly completely, despite its mutations in *ribF*, *mdaB* and *frxA* (D Dailidiene and DE Berg, unpublished data). This result indicates that the *rdxA-wt* (functional) allele is “epistatic” to (masks effect of) several other mutations that contribute to Mtz resistance. To learn if the changes in resistance phenotype conferred by *fur* mutations depended on M1.5′s other resistance-associated mutations, recipient strains were prepared by moving the *Δfur-aphA* allele into the ancestral wild type strain 26695 and into its derivative mutant only in *rdxA* and *frxA* (“strain M2.2”; contains functional alleles of genes *ribF* and *mdaB*). These *Δfur* derivative strains were then transformed with DNA containing informative *cat*-linked *fur* alleles.

Neither the *Δfur* nor *furR3I* alleles markedly affected the very low level of intrinsic Mtz resistance of 26695 wild type (phenoype 1R,3S), in accord with only *rdxA-wt* being epistatic on other resistance gene mutations. In strain M2.2 (null alleles in *rdxA* and *frxA*), however, *furR3I* caused a mild but reproducible increase in resistance (from 32R,50S to 50R,64S). Resistance was also enhanced by a *furT41A* mutation (to 64R,90S), and was sharply diminished by the furH99* nonsense mutation (to 8R,12S). The *furR3S* mutation also decreased resistance in the M2.2 background, albeit rather subtly: like *fur-wt*, *furR3S* allowed 100% cell survival of M2.2 on agar with 32 µg Mtz/ml; most significant, however, on agar with 50 µg Mtz/ml, the *fur-wt* and *furR3S* alelels allowed survival of 10^−1^ and only 10^−5^, respectively. These differences in survival were seen in repeated tests in which these two isogenic strains, grown in parallel on Mtz-free agar, diluted and aliquots of diluted cultures were spotted on complementary halves of the same plates with 50 µg Mtz/ml in the agar.

The generality of effects seen in these 26695 lineage strains was evaluated further using derivatives of reference strains SS1, X47 and G27, each with null alleles of *rdxA* and *frxA* and functional wild type alleles of *ribF* and *mdaB*) (corresponding to the 26695-derived strain M2.2 used above). Resistance was also diminished by the *Δfur, furR3S* and *furH99* alleles and enhanced by the *furR3I* and *furT41A* alleles in these strain backgrounds (data not shown). Collectively, these results indicate that wild type alleles of *ribF* and *mdaB* are not epistatic on *fur* mutant alleles, and that the regulatory imbalances caused by changing *H. pylori* Fur's unique N terminal arm or body are general, not likely to depend on any unique strain genetic background.

## Discussion

*H. pylori*'s Fur protein is a multifunctional regulator that controls transcription of dozens of genes, some negatively and some positively, and variously in response to iron availability, pH and oxidative stress (Fig. 1). The present mutational analysis was begun after finding a mutation affecting *H. pylori* Fur's distinctive N terminal arm after multiple steps of selection for ever higher levels of Mtz resistance, and was motivated by interest in multifunctional regulatory proteins such as Fur and the medical significance of *H. pylori* and its resistance mechanisms. Following a test cross that established that this mutation (*furR3I*) had indeed increased resistance, we made additional *fur* gene mutations and moved them to the *H. pylori* chromosome by PCR and transformation methods, scored them for effects on Fur protein function using a sensitive and efficient assay for changes in Mtz resistance levels, and interpreted their effects in terms of the recently determined structure of zinc-loaded *H. pylori* Fur protein [9]. Prominent among the mutations that increased resistance were seven that affected residues at the interface between Fur protein's N-terminal DNA binding and C-terminal dimerization domains (*furG40D*, *furT41P,I,K,M* and *Q*, and *furE95G*), and that might affect Fur protein's iron-binding, dimer formation or stability, or Apo-Fur or Fe-Fur binding to cognate DNA sites; another resistance mutation altered a DNA binding surface (*furK57T*); and two others also affected Fur's N terminal arm (*furR3K* and *furE8K*) (Fig. 2). Two other *fur* mutations were found recently by others [19] in Mtz resistant clinical isolates (*furC78Y, furP114S*). These mutations seemed to diminish Apo-Fur protein's binding to the *sodB* promoter and thereby to allow increased superoxide dismutase synthesis and detoxification of Mtz's activation products [19].

The ability of Fur to control transcription of many genes when iron-bound and other genes when iron-free is an important feature of this global regulatory protein. Using a PCR-targeted mutation and transformation strategy we randomly changed the codons for five residues that were implicated by structural considerations in iron binding (H42, E90, H97, H99 and E110; Fig. 2). Only nine binding pocket mutations were recovered without other inactivating changes in *fur* among the 62 transformants screened; each such mutation conferred less resistance than did a Δ*fur* allele (Fig. 4C); and two of them (*furH42L* and *furH42V*) caused slow growth. Most other transformants contained additional frameshift or scrambled sequence mutations, implying that most iron binding pocket changes were deleterious or lethal. A strain with one of these putatively lethal mutations (*furH99R*) was readily obtained if the gene also contained a DNA binding site mutation (*furY65N*). Accordingly, we suggest that the apparently lethal or deleterious effects of many mutations and the Mtz sensitization caused by those that were recovered reflect the same phenomenon: disruptions in the normally balanced expression of many Fur-regulated genes that vary in severity, and that in each case stem from decreased iron-dependent regulation without sufficient change in iron-independent (Apo) regulation. Just how iron governs Fur protein's conformation and activity is not yet known, but might entail changes caused by iron binding per se [33], by oxidation of bound iron [34], or by iron-catalyzed protein (histidine) oxidation [35].

Noteworthy in the context of the iron binding pocket point mutations, Mtz resistance was also decreased by nonsense mutations at codons 93, 99 and 115 in the 150 codon *fur* gene. The resultant truncated Fur proteins should retain the N terminal DNA binding domain, but lack the C terminal dimerization domain and a well-structured iron-binding pocket. We suggest that these remnants would retain Apo-Fur activity; and that their binding activity, although weakened by the inability to dimerize, nevertheless should be sufficient to impact on transcription of genes affecting Mtz susceptibility. Nonsense codons inserted closer to *fur*'s 5′ end exhibited the mild decrease in Mtz resistance characteristic of simple null (deletion) alleles, which would be explained if all Fur function is lost in these shorter remnants, and/or if remnant proteins are degraded.

*H. pylori* Fur's distinctive N-terminal arm was identified as part of a well-defined structure in the recently released zinc-loaded Fur protein structure [9], with residue R3 participating in a hydrogen bond network with residues in the DNA-binding domain close to metal-binding residue H42 (Fig. 2). Our *fur* codon 3 mutagenesis results suggest, however, that this is not R3′s only significant interaction – if R3 were important only for stabilizing N-terminal arm-DNA binding domain interactions, all codon 3 mutations that altered Mtz resistance should have changed this phenotype in the same direction. However, two codon 3 mutations increased resistance (*furR3I*, *furR3K*), whereas two others markedly decreased resistance (*furR3S*, *furR3N*) more than did a simple Δ*fur* (*null*) mutation. Mtz resistance was even further diminished when the Mtz-sensitizing alleles *furR3S* or *furR3D* were combined with furH99* (stop) (Fig. 2, 4A,D), which indicates that neither an ability to bind iron nor dimerization is needed for codon 3 mutations to affect Fur protein activity. We propose that the N terminal arm's role in Apo-Fur is distinct from that in Fe-Fur. Precedents from other regulatory proteins [21]–[25] suggest that the Fur arm might also affect Fur regulon gene expression via interaction with target DNAs and/or a cellular effector such as RNA polymerase.

The need for further analysis of just how specific changes in *H. pylori*'s Fur protein affect its various actions and associated phenotypes is also emphasized by a recent study of alanine replacement mutations affecting iron binding pocket residues [18]. The growth of *furE90A*-containing *H. pylori* under iron-replete conditions depressed transcription of the Apo-Fur-repressed iron storage gene *pfr*, whereas transcription of an Apo-Fur-induced gene, *amiE*, was not much elevated, nor did the *furE90A* mutation significantly affect iron-loading of Fur protein in vitro. In addition, a *furE110A* allele, which one might have expected to affect iron-binding similarly had effects distinct from those of *furE90A* on *pfr* and *amiE* gene transcription in vivo and on iron loading and protein dimerization in vitro [18]. These divergent results are in accord with findings of distinct functional roles for residues at different positions in the metal binding pocket of another regulatory metalloprotein [36].

The sensitivity and convenience of Mtz resistance as a diagnostic phenotype and the metabolic changes that particular *H. pylori* Fur alleles elicit, should encourage further studies. We envision (i) further mutant hunts, using other informative phenotypes such as susceptibility to low pH or oxidative stress and expression profiling, expecting that many of the point mutations obtained will have far stronger effects than those conferred by standard gene inactivation; (ii) analyses of mutational effects on Fur activity, transcript profiles, binding to cognate DNA regulatory sites and protein interaction partners, and the structures involved [6], [13], [18], [31]–[37]; (iii) selection for and analysis of compensatory or suppressor mutations within *fur* or elsewhere in the genome, starting with alleles such as *furR3N* or furH99* that decrease resistance [22], [23], [38]; and (iv) placement of *fur* under control of a separately regulated promoter [39], to allow studies of the expression of diverse Fur regulon genes, uncomplicated by Fur regulation of its own expression, and to allow potentially lethal alleles to be recovered efficiently, the immediate consequences of their expression examined, and suppressors of their lethality obtained and characterized.

In conclusion, the simple phenotypic results obtained in our *fur* mutation studies to date, and prospects for informative future analyses illustrate the power of PCR- and chromosomal transformation based mutational analysis of protein structure and function and regulatory circuitry. This strategy should be applicable to many genes of interest in any transformable microbial species.

## Materials and Methods

### Fur protein structure modeling

Molecular graphics images (Fig. 2) were produced using the UCSF Chimera package from the Resource for Biocomputing, Visualization, and Informatics at the University of California, San Francisco (http://www.cgl.ucsf.edu/chimera/docs/credits.html; supported by NIH P41 RR001081). [10] using recently reported *H. pylori* Fur structural data [9].

### *H. pylori* strains and general methods

Most experiments reported here were carried out with Mtz resistant *H. pylori* strain M1.5. This strain had been derived from reference strain 26695 [40] by five sequential cycles of selection for increased resistance, each cycle associated with a new mutation that likely diminished the ability of cells to chemically reduce Mtz from prodrug to bactericidal agent (genes involved were *rdxA, frxA, mdaB, ribF* and *fur*) [20]. Also used were strain 26695 wild type, its *rdxA frxA* mutant derivative M2.2, and equivalent *rdxA, frxA* mutant derivatives of unrelated strains SS1, X47 and G27 [41]–[43] *H. pylori* strains were cultured at 37°C in a standard microaerobic atmosphere (5% O_2_, 10% CO_2_, 85% N_2_) on brain heart infusion-7% horse blood agar plates with 0.4% isovitalex and antibiotics amphotericin B (8 mg/l), trimethoprim (5 mg/l), and vancomycin (6 mg/l). chloramphenicol (15 mg/l) or kanamycin (25 mg/l) were added as needed to select for transformants. Metronidazole (Mtz) was added at various concentrations for quantitative scoring of susceptibility and resistance at concentrations appropriate for the strains being tested, as described [20], [29]. Natural transformation was carried out by adding 7 µl of purified PCR product or 1 µg of genomic DNA to a lawn of cells growing exponentially on nonselective medium, and restreaking the population on selective (generally chloramphenicol containing) medium after 6–8 hrs or overnight incubation to obtain transformant colonies.

### PCR-based construction of strains with random mutations in *fur*

Error prone PCR was carried out in 100 µl volumes containing 20 fmol of genomic DNA from a *fur,cat* (Fig. 3) derivative of strain 26695, 30 pmole of each primer (x5K, x4; Table S1), 5 unit of *Taq* polymerase, 0.2 mM of dGTP, dATP, 1 mM of dCTP, dTTP, 0.5 mM of MnCl_2_ in PCR buffer (1x buffer; 7 mM MgCl_2_, 50 mM KCl, 10 mM Tris-Cl (pH 8.3)). The PCR product was used to transform 26695 *Δfur-aphA*, and chloramphenicol resistant (Cam^r^) transformants were selected, sometimes with accompanying selection for growth on medium with 200 µg of Mtz/ml, as described above.

### PCR-based construction of strains with targeted *fur* mutations

Alleles with mutations targeted to specific sites were constructed by assembling individual PCR products without need for recombinant DNA plasmid cloning. In brief, assembly depends on overlaps of ≥20 bp at the ends of DNAs to be joined together, which, in turn, result from the design of PCR primers used in amplification [27], [28]. To construct several different mutant alleles simultaneously, a forward primer upstream of *fur* (#1 in Fig. 3B; x5K in Table S1) and a reverse mutagenic primer (#2 in Fig. 3B) was used to amplify part of *fur*, and a forward mutagenic primer (#3 in Fig. 3B) and a reverse primer downstream of *cat* (#4 in fig. 3B; x4 in Table S1) was used to generate a complementary part of *fur* and the downstream *cat* gene and adjacent *H. pylori* sequences. A second round of PCR assembly using mixture of these two fragments and primers 1 and 4 yielded a full-length *fur,cat* product likely to be mutant at the site dictated by mutagenic primers #2 and #3. This product was used in transformation (Fig. 3).

### Determination of Mtz resistance phenotypes

Frozen *H. pylori* cultures were streaked onto Mtz-free BHI agar and incubated for 3 days. Then bacterial growth was restreaked on fresh Mtz-free BHI agar and incubated for 1 day. The resulting young exponentially growing cells were suspended in phosphate buffered saline; a series of 10-fold dilutions of these suspensions was prepared; and 10 µl of each dilution was spotted on one half of freshly prepared BHI agar plates containing appropriate concentrations of Mtz. A strain was considered to be susceptible to concentrations of Mtz that decreased its efficiency of colony formation by single cells at least 10 fold. Each such strain was tested at least twice to assess reproducibility, generally along with a different strain that might differ slightly in resistance, and that was spotted in parallel on the other half of the same plate. This protocol for quantitative and reproducible determination of levels of Mtz susceptibility or resistance, uncomplicated by Mtz's mutagenic action [26], has been detailed elsewhere [20], [29].

## Supporting Information

Table S1 Primer sequences used for *H. pylori fur* gene manipulation and analysis.
(DOC)
