# Supplementary material for: Point Mutations in Helicobacter pylori's fur Regulatory Gene that Alter Resistance to Metronidazole, a Prodrug Activated by Chemical Reduction
Source: PLoS One. 2011 Mar 25;6(3):e18236. doi: 10.1371/journal.pone.0018236 (PMC3064673; doi:10.1371/journal.pone.0018236)
Supplement: Table S1 — Primer sequences used for H. pylori fur gene manipulation and analysis. (DOC) [file pone.0018236.s001.doc]

Table S1. Primer sequences used for *H. pylori fur* gene manipulation and analysis

| **Target site** | Primer name | **Strain 26695 genome sequence coordinates (5’ →3’)** | **sequence**  **(5’→3’)** |
| --- | --- | --- | --- |
| Flanking region | x5k-F | 1089716-1089740 | cct taa ttt agc cgc ttc ttg ttt g |
|  | x4-R | 1091135-1091115 | ctg tag agt tgc ctg gaa ttt gtc a |
| For *fur* sequencing | 1027 | 1090019-1090040 | ccttttagaatggcttgataag |
| *fur*-cam | c1x2 | 1090706-1090682 | *atc cac ttt tca atc tat atc* cca tta aag ata gcc cta tct aag c |
|  | c2x3 | 1090682-1090706 | *ccc agt ttg tcg cac tga taa* gct tag ata ggg cta tct tta atg g |
| ∆ *fur*-kan | kanF x6 | 1090210-1090184 | *atg gtt cgc tgg gtt tat c*ct gat atc ttc ctt atc cgt aaa atg a |
|  | aphaR x3 | 1090682-1090706 | *tta ctg gat gaa ttg ttt tag tac* cgc tta gat agg gct atc ttt aat gg |
| Arg3 random | R3n-F | 1090193-1090239 | gga taa gga aga tat cag cat gaa a***NN*** ***B***tt aga aac ttt gga atc ca |
|  | R3n-R | 1090239-1090193 | tgg att cca aag ttt cta a***VN*** ***N***tt tca tgc tga tat ctt cct tat cc |
| ∆ codon2-7 | d27-F | 1090192-1090214/1090233-1090255 | cgg ata agg aag ata tca gca tgg aat cca ttt tag agc gct tga gg |
|  | d27-R | 1090249-1090233/1090214-1090190 | cgc tct aaa atg gat tcc atg ctg ata tct tcc tta tcc gta |
| Thr41 | T41n-F | 1090312-1090352 | gcg ttt tgt atc gca gcg gc***N*** ***NB***c acc taa gcc ctg aag aa |
|  | T41n-R | 1090352-1090317 | gcg tga ttt ctt cag ggc tta ggt g***VN*** ***N***gc cgc tgc gat aca a |
| Correct T41A | T41T-F | 1090312-1090352 | gcg ttt tgt atc gca gcg gc***a ca***c acc taa gcc ctg aag aa |
|  | T41T-R | 1090352-1090317 | gcg tga ttt ctt cag ggc tta ggt g***tg t***gc cgc tgc gat aca a |
| His42 | H42L-F | 1090318-1090356 | tgt atc gca gcg gca ca***t*** ***ta***c taa gcc ctg aag aaa tca |
|  | H42A-R | 1090357-1090317 | gtg att tct tca ggg ctt ag***a*** ***gc***t gtg ccg ctg cga tac aa |
|  | H42n-F | 1090318-1090356 | tgt atc gca gcg gca ca***N*** ***NB***c taa gcc ctg aag aaa tca |
|  | H42n-R | 1090357-1090317 | gtg att tct tca ggg ctt ag***V*** ***NN*** t gtg ccg ctg cga tac aa |
| Tyr65/Arg66 | Y65N-F | 1090387-1090430 | cta gca ttt ctt cag tc***a* *at***c gca ttt tga att tct tag aaa aag |
|  | R66M-R | 1090430-1090387 | ctt ttt cta aga aat tca aaa t***ca* *t***at aga ctg aag aaa tgc tag |
| Tyr65 | Y65L-F | 1090387-1090430 | cta gca ttt ctt cag tc***t*** ***ta***c gca ttt tga att tct tag aaa aag |
|  | Y65L-R | 1090430-1090387 | ctt ttt cta aga aat tca aaa tgc g***ta*** ***a***ga ctg aag aaa tgc tag |
| Glu90 | E90A-F | 1090467-1090498 | ggt cgg cgc tat ***gct*** att gcg cgt aaa gaa ca |
|  | E90n-R | 1090494-1090463 | ctt tag ccg caa t***VNN***at agc gcc gac cgc tt |
| His97 | H97R-F | 1090486-1090517 | cgg cta aag aac acc gcg atc aca tca ttt gt |
|  | H97n-R | 1090516-1090483 | caa atg atg tga tc***VNN***g tgt tct tta gcc gca a |
| His99 | H99R-F | 1090487-1090523 | ggc taa aga aca cca tga t***cg*** ***c***at cat ttg ttt gca t |
|  | H99R-R | 1090527-1090490 | cgc aat gca aac aaa tga t***gc*** ***g***at cat ggt gtt ctt ta |
|  | H99n-F | 1090487-1090523 | ggc taa aga aca cca tga t***NN*** ***B***at cat ttg ttt gca t |
|  | H99n-R | 1090527-1090490 | cgc aat gca aac aaa tga t***VN*** ***N***at cat ggt gtt ctt ta |
| To separate H99, H82 ETC | H99d-F | 1090469-1090496 | tcg gcg cta tga aat tgc ggc taa aga a |
|  | H99d-R | 1090501-1090477 | tgg tgt tct tta gcc gca att tca t |
| Glu110 | E110R-F | 1090520-1090555 | gca ttg cgg taa gat cat t***cg*** ***c***tt tgc aga ccc tga |
|  | E110n-R | 1090554-1090519 | cag ggt ctg caa a***VN*** ***n***aa tga tct tac cgc aat gca |
|  |  |  |  |
| Lys57 | K57stop-F | 1090362-1090400 | tct atc cgc caa aag gac ***taa*** aac act agc att tct tca |
|  | K57stop-R | 1090400-1090362 | tga aga aat gct agt gtt ***tta*** gtc ctt ttg gcg gat aga |
| Cys78 | C78stop-F | 1090405-1090464 | atc gca ttt tga att tct tag aa aag aaa att tta tc***t*** ***ga***g ttt tag aaa ctt caa aaa gcg gtc ggc gct a |
|  | C78stop-R | 1090477-1090418 | tag cgc cga ccg ctt ttt gaa gtt tct aaa ac***t*** ***ca***g ata aaa ttt tct ttt tct aag aaa ttc aaa atg cga t |
| Gly86 | G86stop-F | 1090449-1090491 | tta gaa act tca aaa agc ***tga*** cgg cgc tat gaa att gcg gct a |
|  | G86stop-R | 1090491-1090449 | tag ccg caa ttt cat agc gcc g***tc*** ***a***gc ttt ttg aag ttt cta a |
| Ala93 | A93stop-F | 1090469-1090509 | tcg gcg cta tga aat tgc c***ta a***aa aga aca cca tga tca ca |
|  | A93stop-R | 1090509-1090469 | tgt gat cat ggt gtt ctt t***tt*** ***a***cg caa ttt cat agc gcc ga |
